# Supplementary figures and images for: COVID-19: A complex disease with a unique metabolic signature
Source: PLoS Pathog. 2023 Nov 9;19(11):e1011787. doi: 10.1371/journal.ppat.1011787 (PMC10662774; doi:10.1371/journal.ppat.1011787)

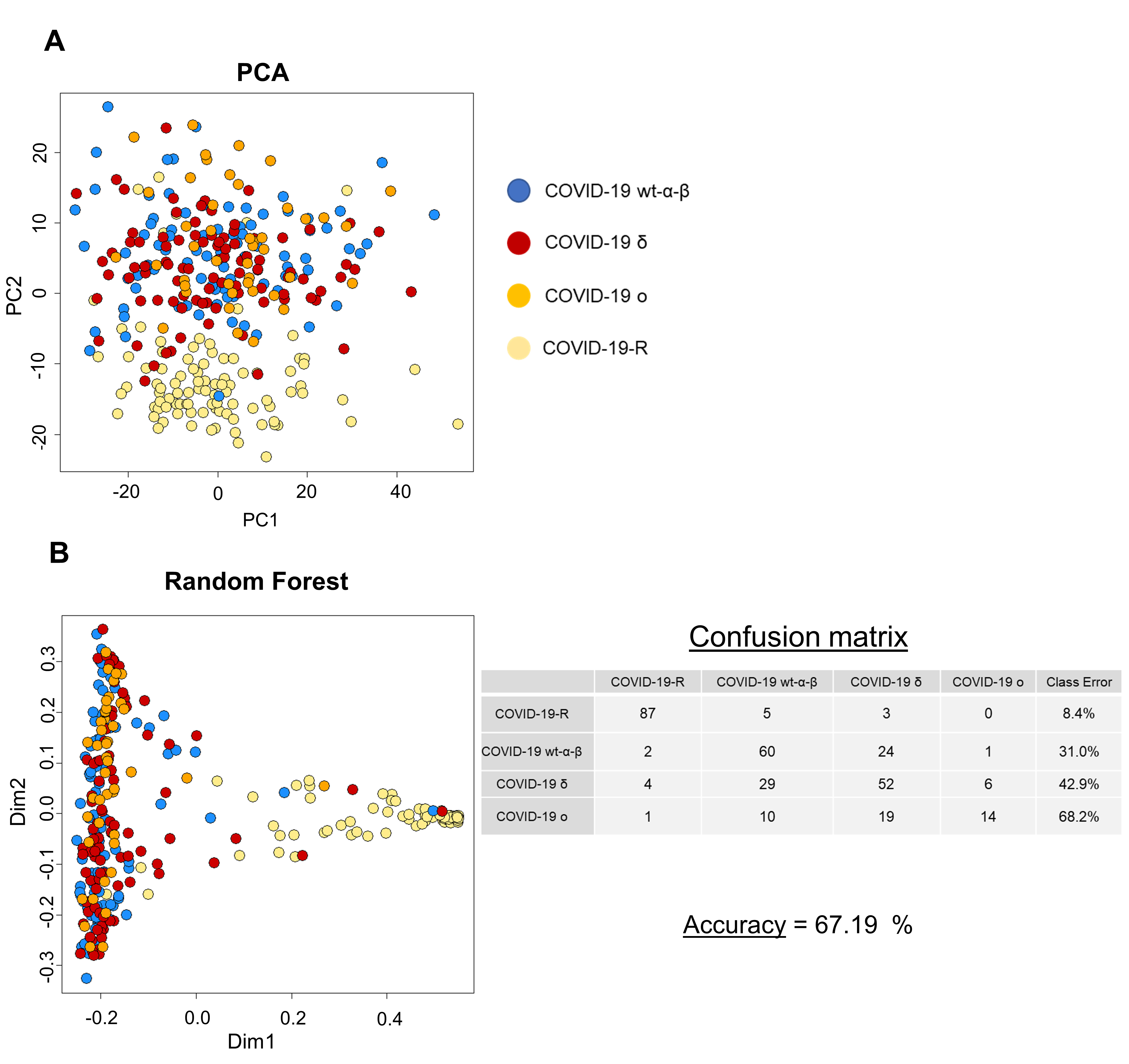

Supplement: S1 Fig — A) PCA Score Plot based on bucketed NOESY spectra of the main three COVID-19 variant groups and COVID-19-R group. B) Proximity plots of the RF model discriminating the COVID-19 variant groups and COVID-19-R subjects using bucketed NOESY spectra. The confusion matrix and the accuracy value are reported. Colour coding: wt-α-β group (cyan); δ group (red); o group (orange); COVID-19-R (yellow). (TIF) [file ppat.1011787.s005.tif]

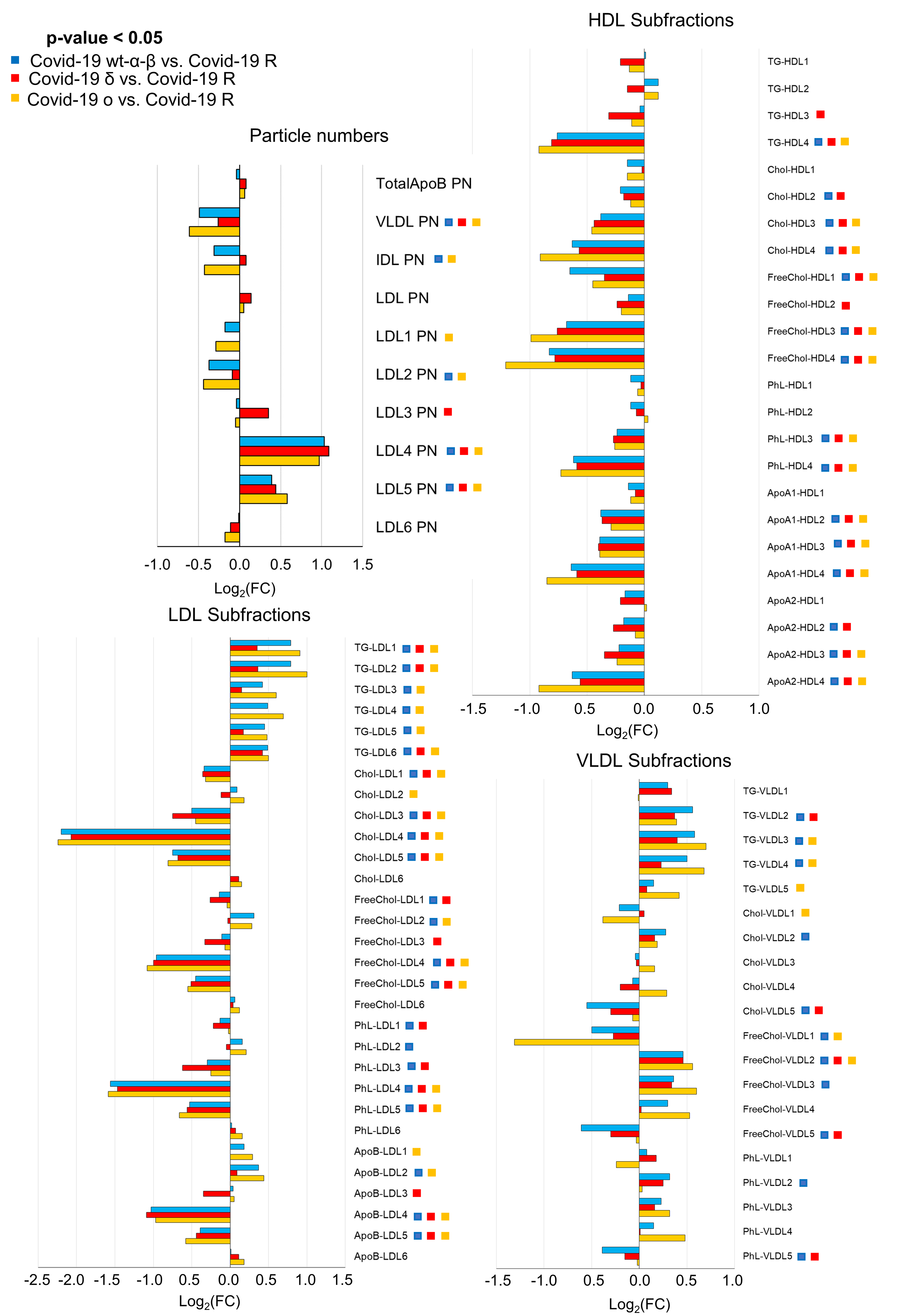

Supplement: S2 Fig — Values of Log2 fold change (FC) of quantified lipoprotein parameters (particle numbers and HDL, LDL, and VLDL subfractions). Positive/negative values have higher/lower concentration in plasma samples from each of the three variant COVID-19 groups with respect to COVID-19-R group. p-values <0.05 are highlighted with colored squares. Colour coding: wt-α-β group (cyan); δ group (red); o group (yellow). (TIF) [file ppat.1011787.s006.tif]

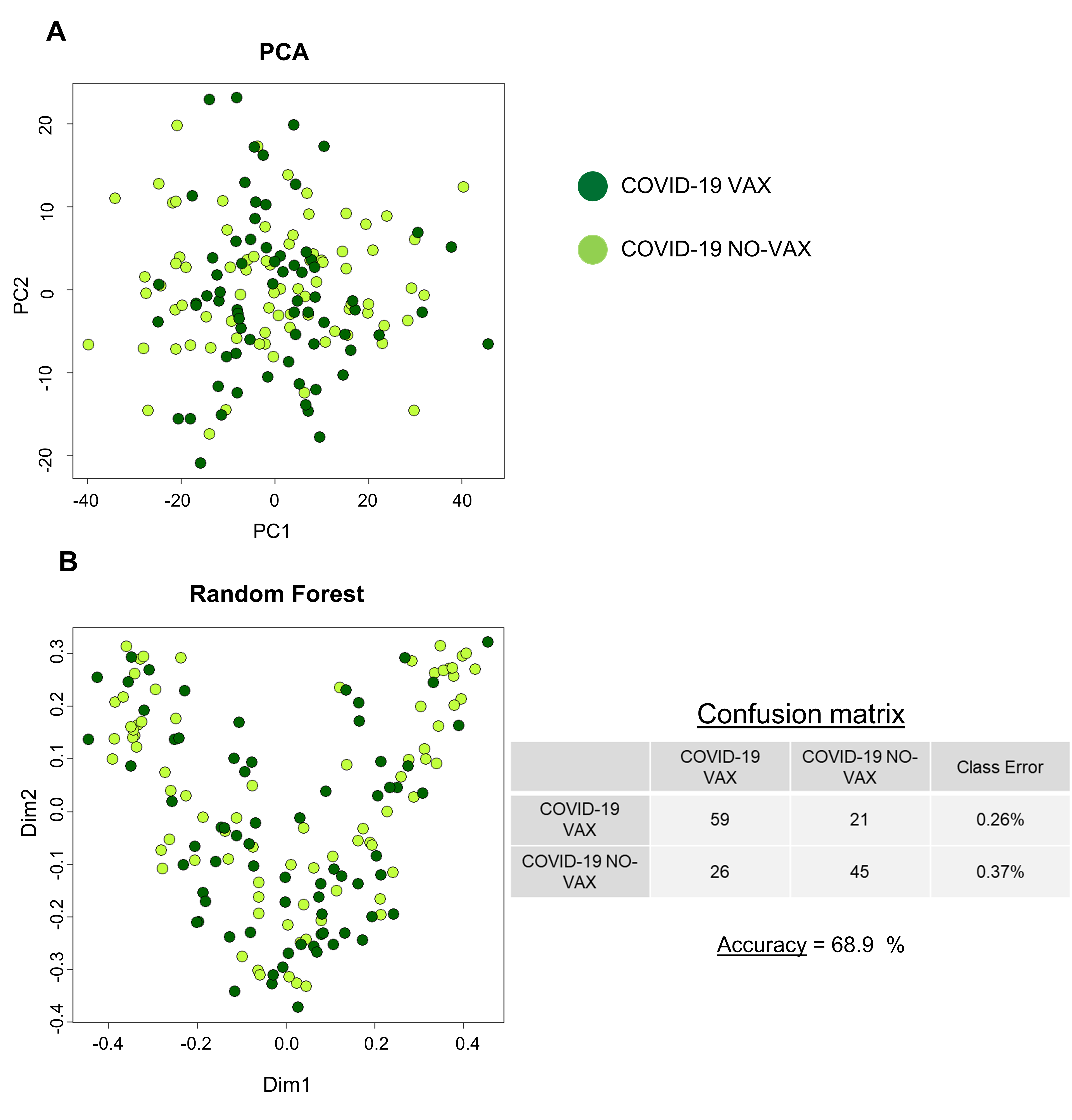

Supplement: S3 Fig — A) PCA Score Plot based on bucketed NOESY spectra. B) Proximity plots of the RF model discriminating the COVID-19 VAX group and the COVID-19 NO-VAX group using bucketed NOESY spectra. The confusion matrix and the accuracy value are reported. Colour coding: VAX group (green); NO-VAX group (grey). (TIF) [file ppat.1011787.s007.tif]

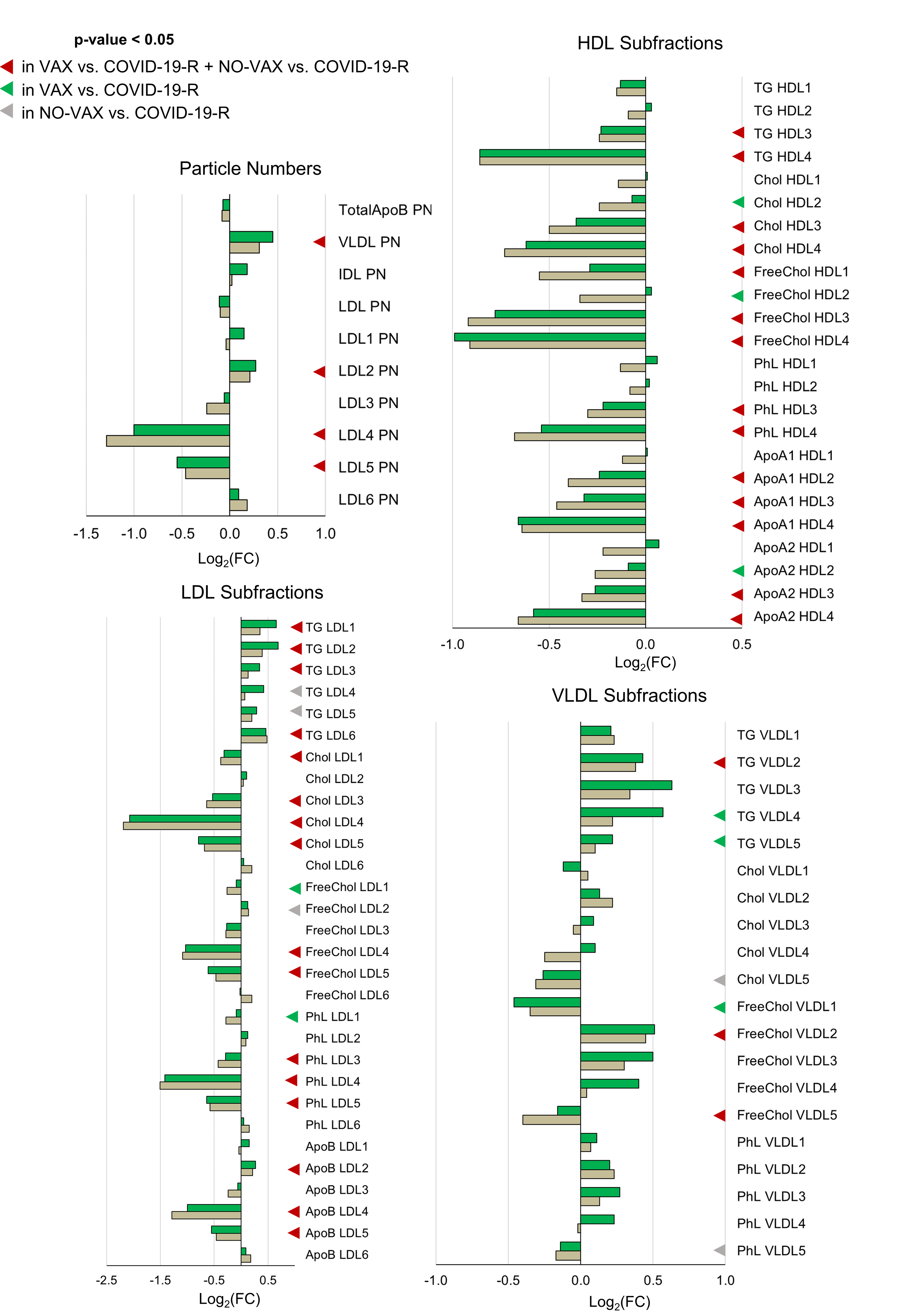

Supplement: S4 Fig — Values of Log2 fold change (FC) of quantified lipoprotein parameters (particle numbers and HDL, LDL, and VLDL subfractions). Positive/negative values have higher/lower concentration in plasma samples from the VAX or NO-VAX groups with respect to the COVID-19-R group; p-values <0.05 are highlighted with coloured triangles. Colour coding: VAX group (green); NO-VAX (grey). (TIF) [file ppat.1011787.s008.tif]

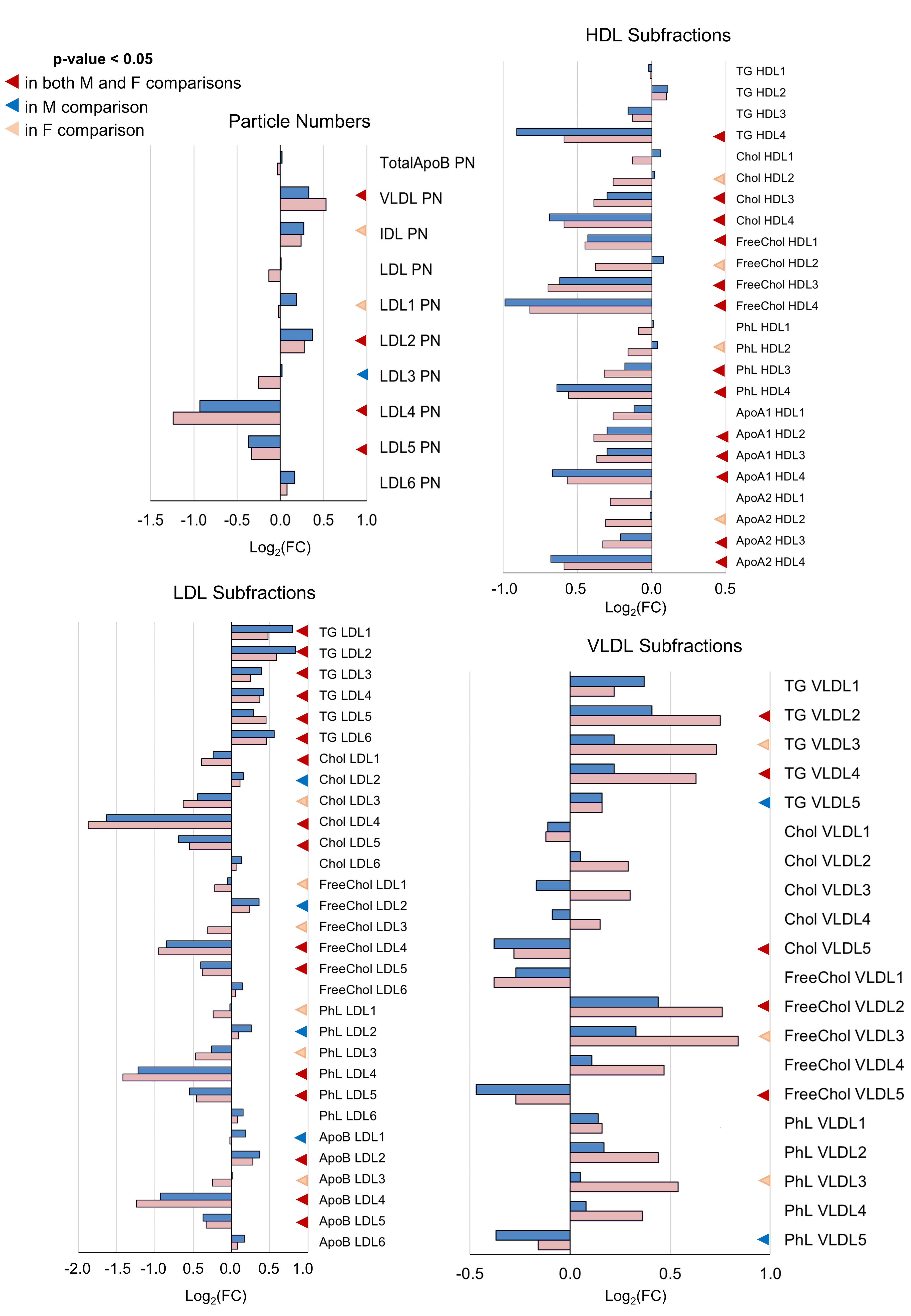

Supplement: S5 Fig — Values of Log2 fold change (FC) of quantified lipoprotein parameters (particle numbers and HDL, LDL, and VLDL subfractions). Positive/negative values have higher/lower concentration in plasma samples from male (M) or female (F) groups with respect to M or F COVID-19-R subjects, respectively; p-values <0.05 are highlighted with coloured triangles. Colour coding: male group (blue); female group (pink). (TIF) [file ppat.1011787.s009.tif]

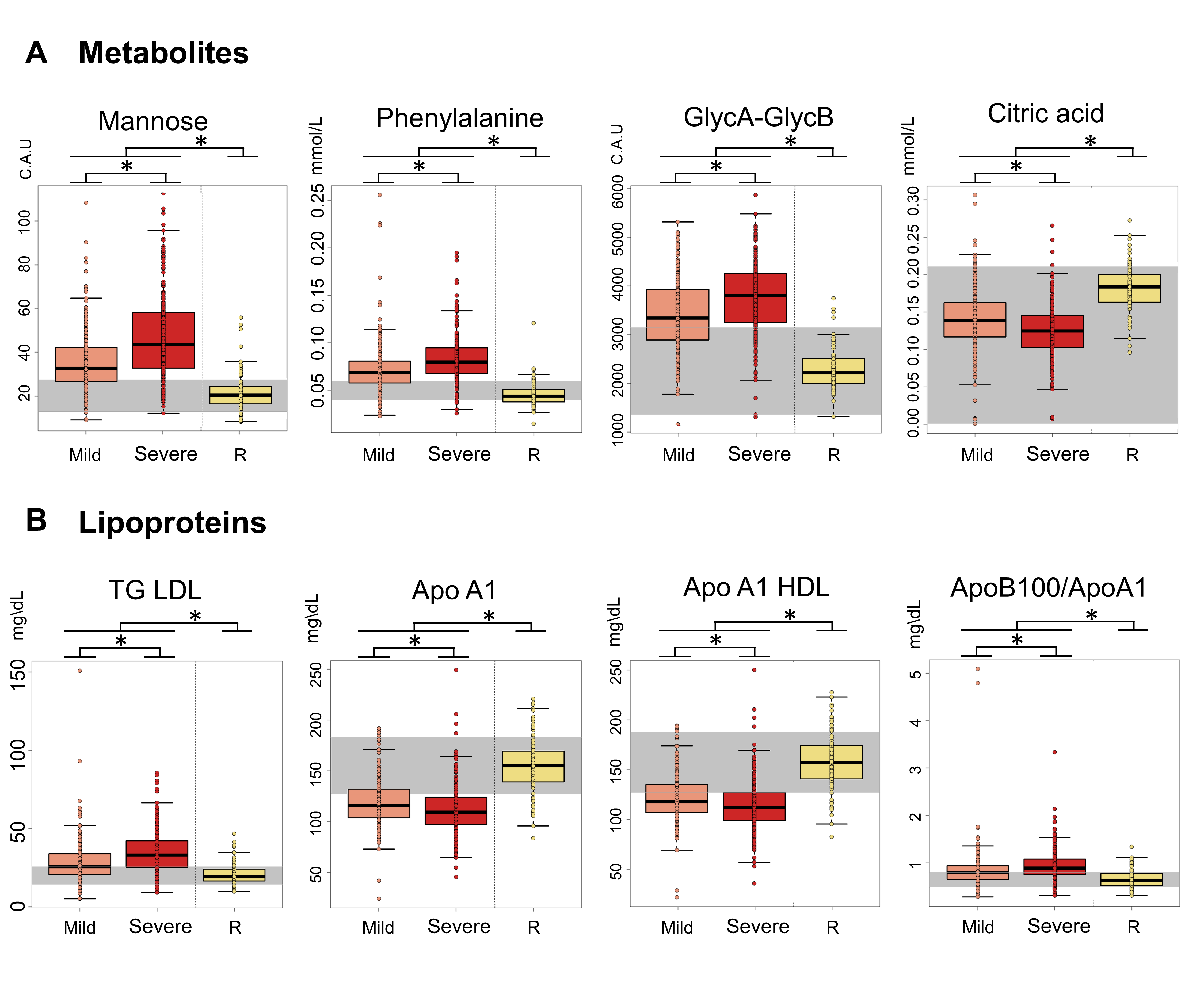

Supplement: S6 Fig — Box plots of the concentration levels for (A) metabolites and (B) lipoprotein parameters (main parameters, calculated figures and main fractions) that have a p-value < 0.05 and a large Cliff’s delta effect size in the comparison between COVID-19 and COVID-19-R groups and whose levels are also significantly altered between mild and severe patients. In each plot, the grey stripe embraces the concentration range in the reference “healthy” population. Colour coding: mild (light red); severe (red); COVID-19-R (yellow). *indicates p-value < 0.05: the upper line indicates the statistical significance between all the COVID-19 subjects and the COVID-19-R group; the lower line indicates statistical significance between mild and severe subjects. (TIF) [file ppat.1011787.s010.tif]

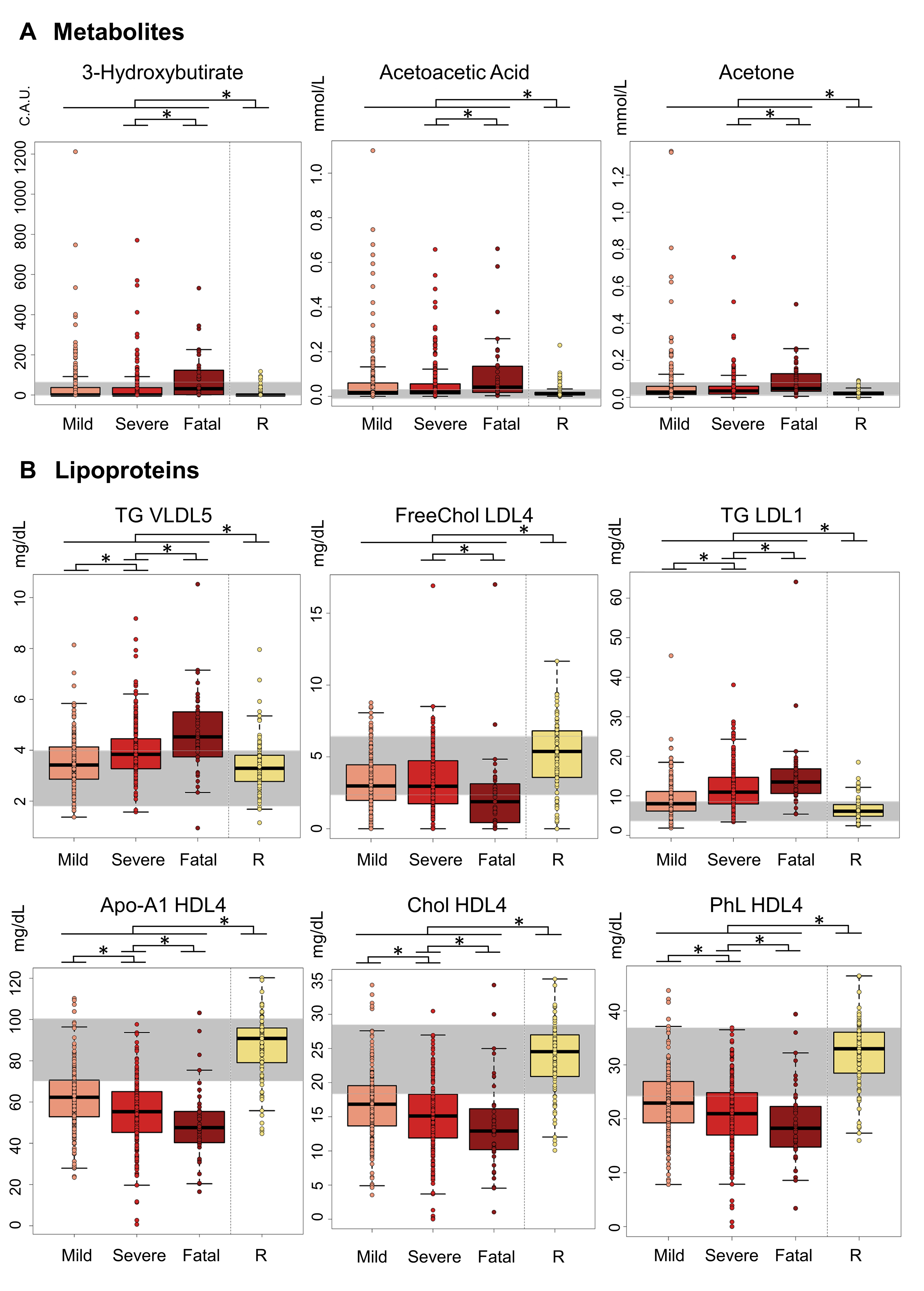

Supplement: S7 Fig — Box plots of (A) metabolites and (B) lipoproteins concentration levels in COVID-19 positive subjects grouped according to disease severity. The concentration levels in COVID-19-R subjects are also reported. Colour coding: mild (light red); severe (red); fatal (dark red); recovered subjects (yellow). * indicates p-value < 0.05: the upper line indicates the statistical significance between all the COVID-19 subjects and the COVID-19-R group; the lower lines indicate statistical significance between pairs of severity groups. (TIF) [file ppat.1011787.s011.tif]

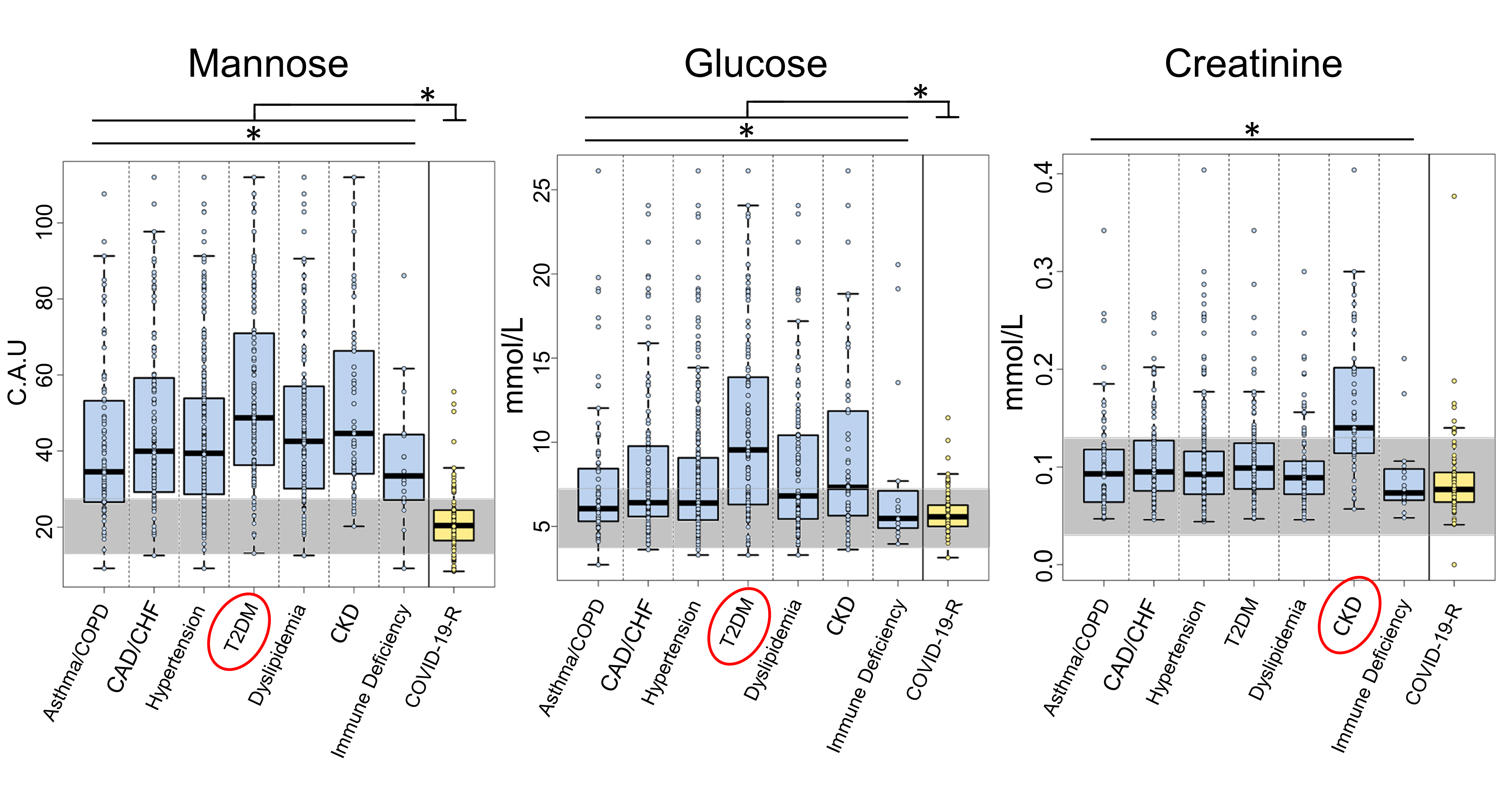

Supplement: S8 Fig — Box plots of Mannose, Glucose and Creatinine concentration levels in the 510 COVID-19 positive subjects grouped as a function of the main comorbidities. The concentration levels in COVID-19-R subjects are also reported as control values (yellow bar). In each plot, the grey stripe covers the concentration range in a "healthy" population. * indicates p-value < 0.05: the upper line indicates the statistical significance between all the COVID-19 subjects and the COVID-19-R group; the lower line indicates that at least one of the comorbidity groups (circled in red) is significantly different from all the others. List of abbreviations: COPD: Chronic Obstructive Pulmonary Disease; CAD: Coronary Artery Disease; CHF: Congestive Heart Failure; T2DM: Type 2 Diabetes; CKD: Chronic Kidney Disease. (TIF) [file ppat.1011787.s012.tif]

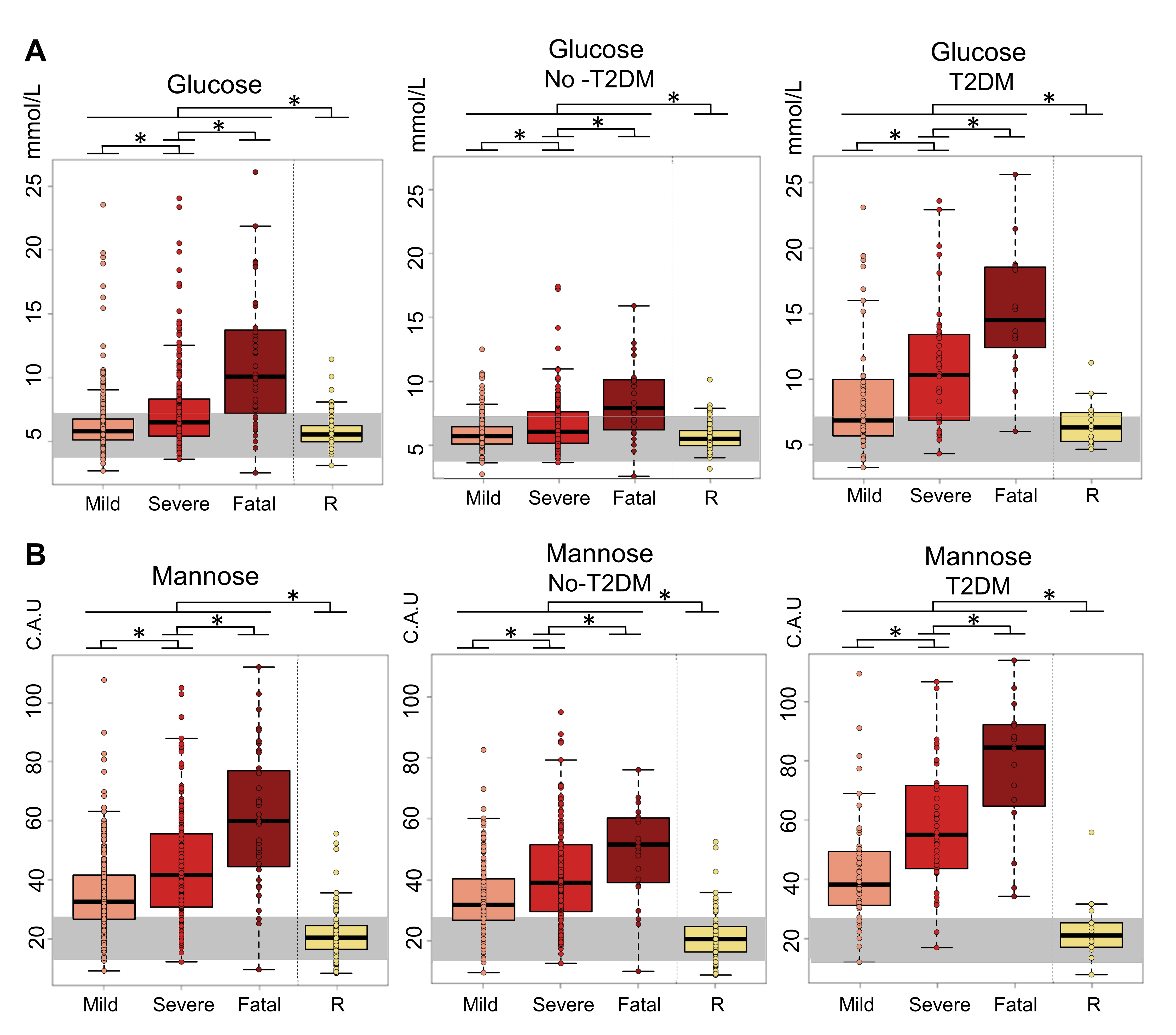

Supplement: S9 Fig — Box plots of (A) mannose and (B) glucose concentration levels in COVID-19 positive subjects grouped according to the grade of the disease severity. The concentration levels in COVID-19-R subjects are also reported. Left panels: all the subjects; middle panels: the T2DM subjects were excluded from the analysis; right panels: only T2DM subjects. In each plot the grey stripe covers the concentration range in a "healthy" population. Colour coding: mild (light red); severe (red); fatal (dark red); recovered subjects (yellow). * indicates p-value < 0.05: the upper line indicates the statistical significance between all the COVID-19 subjects and the COVID-19-R group; the lower lines indicate statistical significance between pairs of severity groups. (TIF) [file ppat.1011787.s013.tif]

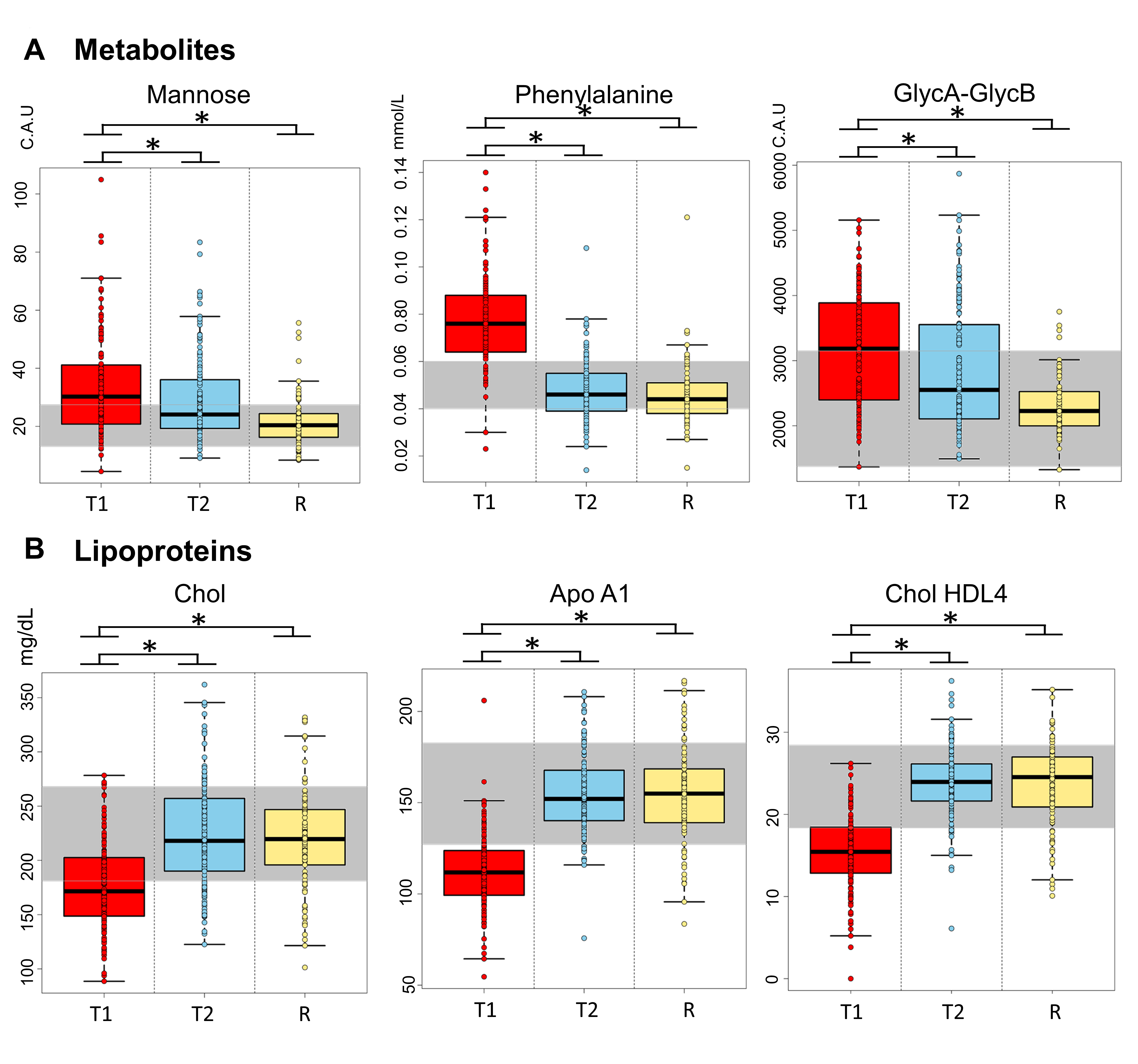

Supplement: S10 Fig — Box plots of the concentration levels for (A) metabolites and (B) lipoproteins that have a p-value < 0.05 and a large Cliff’s delta effect size in the comparison between the plasma samples collected at the moment of the acute infection (T1) and the samples collected at the follow-up visit (T2). The concentration levels in COVID-19-R subjects are also reported as control values. In each plot, the grey stripe embraces the concentration range in the reference “healthy” population. Colour coding: T1 (red); T2 (blue); COVID-19-R (yellow). * indicates p-value < 0.05: the upper line indicates the statistical significance between all the COVID-19 subjects and the COVID-19-R group; the lower line indicates statistical significance between T1 and T2. (TIF) [file ppat.1011787.s014.tif]

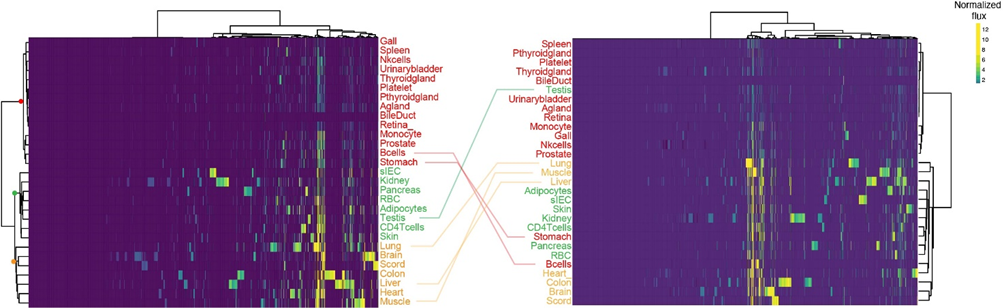

Supplement: S11 Fig — Heatmaps showing the clustering of the different organs according to the activity of each reaction (left severe, right fatal state). Each column represents a reaction in the human metabolic network and its color accounts for the (normalized) activity of that specific reaction in the corresponding organ. In the heatmap on the left, three main clusters are labelled with a red, green and orange dot and the organs belonging to each cluster are labelled accordingly and this color code is maintained in the heatmap on the right. Organs changing clusters in the two heatmaps are connected with a line. (TIF) [file ppat.1011787.s015.tif]

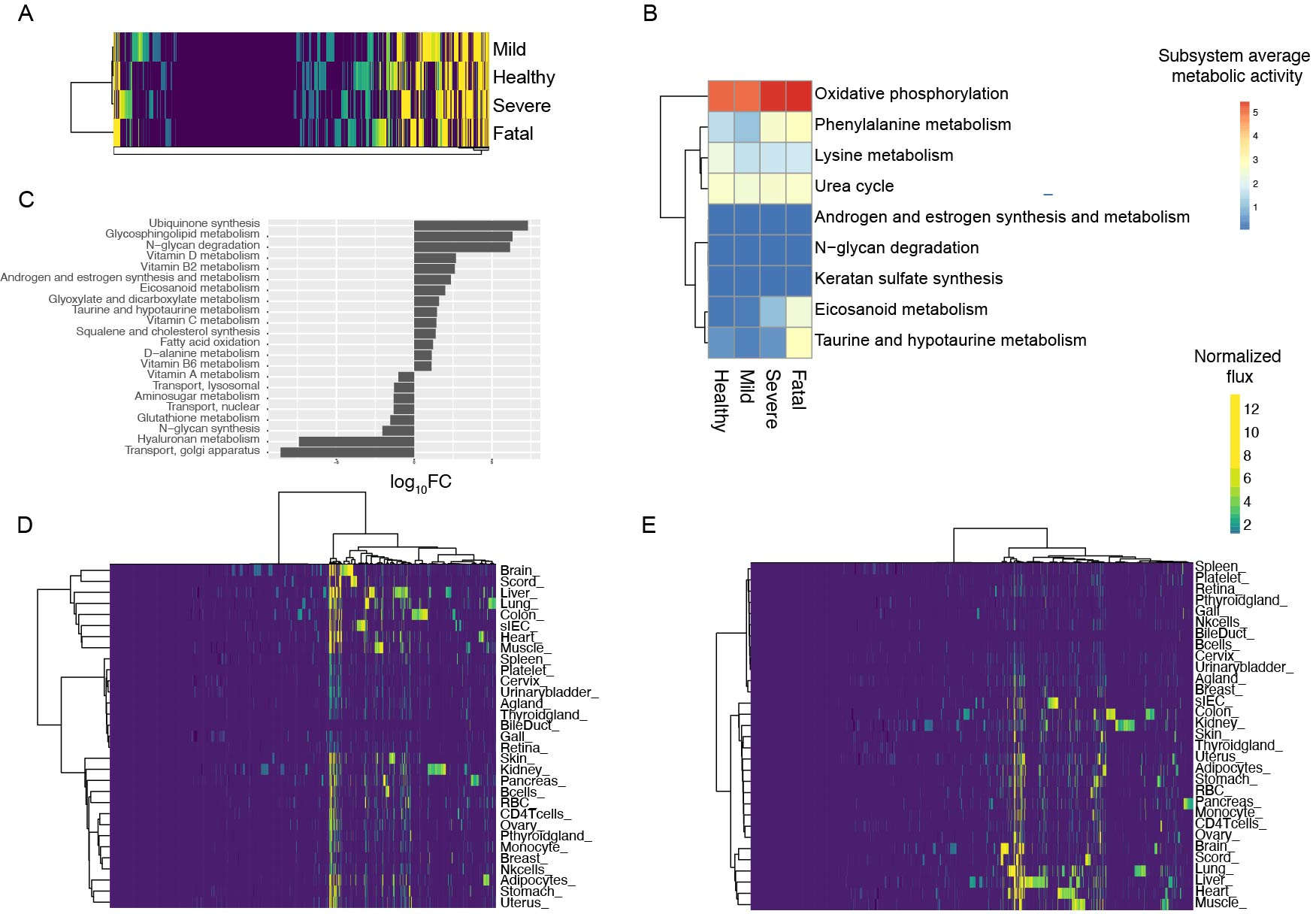

Supplement: S12 Fig — A) Clustering of context-specific models. Each column represents a reaction in the human metabolic network and its color accounts for the (normalized) activity of that specific reaction in the corresponding context-specific model. Models are clustered according to their profile of active/inactive reactions. B) Heatmap that includes those metabolic processes (subsystems) showing an overall flux increase that paralleled that of disease severity (from mild to severe). Their activity in the healthy-model is also included in the figure for clarity. C) Fold change of average metabolic activity for each subsystem in the healthy vs. fatal condition. D-E) Heatmaps showing the clustering of the different organs according to the activity of each reaction (D- healthy state, E- fatal state). As in in the panel A, each column represents a reaction in the human metabolic network and its color accounts for the (normalized) activity of that specific reaction in the corresponding organ. (TIF) [file ppat.1011787.s016.tif]
