# Supplementary material for: COVID-19: A complex disease with a unique metabolic signature
Source: PLoS Pathog. 2023 Nov 9;19(11):e1011787. doi: 10.1371/journal.ppat.1011787 (PMC10662774; doi:10.1371/journal.ppat.1011787)
Supplement: Attachment — Submitted filename: Lettera_FINAL_021023.docx [file ppat.1011787.s017.docx]

 Reviewer's Responses to Questions

**Part I – Summary**

Please use this section to discuss strengths/weaknesses of study, novelty/significance, general execution and scholarship.

 Reviewer #1: In this manuscript, Ghini et. al. investigate the metabolic signature of SARS-CoV-2 infection, in relation to viral variants, vaccination status, sex differences and comorbidities and try to identify metabolites as prognostic markers to the fatal outcome of the disease. Finally, by utilizing a multi-organ metabolic model they attempt to simulate the impact of COVID-19 on the entire host metabolism. The authors try to shed more light on the effects of the SARS-CoV-2 infection on host metabolism and improve our understanding of the pathology of COVID-19. The fact that the authors collected samples from a big cohort and through a broad time period strengthens the study. On the other hand, by employing NMR analysis instead of MS they limit the detection of metabolites and are not able to address all the changes in host metabolism. Overall, the concept of the study is interesting as it addresses understudied aspects of the SARS-CoV-2 infection such as viral variants and vaccination effect. However, there are there are some issues on this study that need to be addressed, in order to improve the quality of the manuscript.

**We thank the Reviewer for his analysis of our manuscript. It is true that the number of metabolites detectable by NMR is smaller than that measured by MS. On the other hand, NMR has the advantage of being highly reproducible and enable an untargeted approach, which not only involves metabolites but also a large ensemble of lipoprotein parameters that has no counterpart in MS studies. We have better specified this point at the beginning of the Discussion (lines 317-320).**

Reviewer #2: The manuscript "COVID-19: a complex disease with a unique metabolic signature" by Ghini et al provide novel dataset describing metabolite and lipoprotein signature in realtion to COVID-19 infection. This is a follow up manuscript to previous work published by Authors. The number of samples used for analysis are good and Authors have provided different comparisons like Sex differences, vaccination, etc. Overall this manuscript provides novel information to the field.

**We thank the Reviewer for these positive comments.**

**Part II – Major Issues: Key Experiments Required for Acceptance**

Please use this section to detail the key new experiments or modifications of existing experiments that should be absolutely required to validate study conclusions.

Generally, there should be no more than 3 such required experiments or major modifications for a "Major Revision" recommendation. If more than 3 experiments are necessary to validate the study conclusions, then you are encouraged to recommend "Reject".

Reviewer #1: 1) In the manuscript it is stated that the samples were obtained at the peak of infection, but it is not clear whether the patients were hospitalized before or after the sampling. Hospitalization and maybe nutrient supplementation, especially for the severe patients and those with fatal outcome, could affect the metabolic signature and in particular the glucose levels. The authors should clarify that the patients received no nutrient supplementation prior to obtaining samples.

**We thank the Reviewer for these comments, that allow us to better specify the recruitment procedure. Blood samples have been collected at the moment of admission to the hospital, when patients were swabbed regardless of the cause of hospitalization, and before the start of any therapy or other interventions (oxygen mask, ventilation, parenteral nutrition). This information is now provided at lines 447-449.**

2) In figure 8, the authors compare the metabolic activity of fatal patients to healthy controls. Wouldn’t a comparison between fatal and severe (recovered) patients would also give us more indications on which metabolic pathways or organs are more perturbed and could lead to death because of SARS-CoV-2 infection?

**As now better specified at lines 521-522, as for the rest of this study and in accordance to previous findings [Ghini V., et al. 2022, PLOS Pathogens, 18(4), e1010443], the recovered subjects are taken as healthy controls.**

**Following the Reviewer’s suggestion, we have also prepared a new figure (Figure S11), where heatmaps are reported showing the clustering of the different organs according to the activity of the reaction in the comparison between severe and fatal states. This new analysis shows that the main contribution to the metabolic dysregulation is associated to the progression from severe illness to fatal event (lines 297-299).**

Reviewer #2: This work is novel but is very similar to what authors have done previously with ~350 samples. The analysis done are different but the techniques used is similar. So, in future authors should make an effort to make this work comprehensive and with more samples and long duration follow up of the patients if possible.

**The Reviewer should take into account that the present and previous studies are associated to a project financed by the region of Tuscany, which was submitted in July 2020. Initially we focused on analyzing the disease, without being able to predict that it would evolve in various waves caused by different variants, nor could we expect the massive vaccination campaign that took place starting from Spring 2021. The first study [Ghini V., et al. 2022, PLOS Pathogens, 18(4), e1010443] is in fact limited to patients enrolled during the period June 2020-April 2021, and all unvaccinated.**

**The second (also associated with an extension of the project granted in the light of the evolution of the pandemic) integrates with the other variants and also involves** **subjects vaccinated with two or more doses (see lines 138-139).**

**Part III – Minor Issues: Editorial and Data Presentation Modifications**
Please use this section for editorial suggestions as well as relatively minor modifications of existing data that would enhance clarity.

Reviewer #1: 1) In the text for figures 1 and 2 the authors state that there are 11/25 metabolites and 16/30 lipoproteins significantly different compared to the control group. But in the figures, the highlighted number of significantly altered metabolites and lipoproteins is different (for example for variant o there are 16 metabolites and 18 lipoproteins highlighted). Why is that? Are the authors only referring to the metabolites changed in all variant groups? That should be made clear.

**The interpretation of the Reviewer is correct; we have rephrased the text at lines 114-116 to make it clearer.**

2) In figure 1, Ornithine is only changed in the o variant group. In the study by Li et. al. (Frontiers in Immunology, 2021) ornithine was found upregulated and correlating positively with inflammation in severe COVID-19 patients. The authors should comment on that, also regarding maybe the less severity of the o variant compared to previous variants of SARS-CoV-2?

**The only article by Li et al. in Frontiers in Immunology, 2021 is a Review article (title: Hyperinflammatory Immune Response and COVID-19: A Double Edged Sword) that does not mention ornithine.**

**In other papers the information about ornithine is not conclusive and often contradictory.**

**For example, a MS study published in Front. Microbiol., [Li T. et al.** **Front Microbiol 2021, 12, 723818], reports that "*metabolites of the ornithine cycle, were significantly higher in severe patients than in mild ones, mainly on days 1–3 or 4–6 since onset*". Another paper reports a decreased ornithine in samples collected in 2021 with respect to samples collected in 2020 [Gil-Redondo R. et al., Metabolites 2022, 12(12), 1206]; no data on omicron variant are reported. In other studies [Bruzzone C. et al., iScience 2020, 23, 101645; Bizkarguenaga M. et al., NMR Biomed 2021, e4637], which still deal with non-omicron variants, ornithine was found to decrease in infected patients.**

**In our case, ornithine is found to increase in wt and to decrease in δ and ο , but this change is found to be significant only in the ο variant. When analysing the log_2_(FC) of ornithine as a function of severity, no meaningful trends were observed.**

3) On the same context, all the ketone bodies are also more increased in the o variant group compared to other groups. The authors should also comment on that.

**It is true that the log_2_(FC) of ketone bodies is higher for the omicron with respect to the other variants, although this value is consistently very high in all cases. According to our findings, ketone bodies are particularly high in fatal cases and the number of fatal cases in the omicron is larger than for the other variants (see line 199). A comment has been added at line 333: "The larger increase in ketone bodies observed for the ο variant can be attributed to the higher percentage of fatal events in this subgroup.".**

4) In figure 3, although the colors are explained in the figure legend, it would be helpful to have a color-coding legend in the figure for VAX and NO-VAX groups.

**Thanks for pointing this out. A new version of the figure has been added, which provides the color coding with the same style used for the other figures.**

5) In line 282 it is written that “This analysis revealed a clear cut between…” Something is missing.

**The sentence is correct in its original form.**

Reviewer #2: Please confirm and update that samples used were >500 and not >600 as the author summary section says >600.

**We have better explained the concept in the author summary (lines 26-27): the identification of dysregulated metabolites in the acute phase (510 patients) emerges from the comparison with the recovered subjects (95 individuals), i.e. 605 independent subjects.**

**The number of samples used is even larger, with 154 individuals out of 510 sampled in a follow-up visit.**

**All these figures are detailed at the beginning of the Results section (lines 89-97).**
